# A second monoclinic polymorph of 6-amino-1,3-dimethyl-5-[(*E*)-2-(methyl­sulfan­yl)benzyl­idene­amino]­pyrimidine-2,4(1*H*,3*H*)-dione

**DOI:** 10.1107/S1600536811027322

**Published:** 2011-07-13

**Authors:** Irvin Booysen, Ismail Muhammed, Anna Soares, Thomas Gerber, Eric Hosten, Richard Betz

**Affiliations:** a University of Kwazulu-Natal, School of Chemistry, Private Bag X01, Scottsville 3209, Pietermaritzburg, South Africa; b Nelson Mandela Metropolitan University, Summerstrand Campus, Department of Chemistry, University Way, Summerstrand, PO Box 77000, Port Elizabeth 6031, South Africa

## Abstract

A new monoclinic form of the title compound, C_14_H_16_N_4_O_2_S, has been identified unexpectedly during an attempt to synthesize a coordination compound. The heterocyclic ring is essentially planar (r.m.s. deviation = 0.005 Å) and makes a dihedral angle of 8.77 (5)° with the benzene ring. This is in contrast to 12.24 (7)° reported for the first monoclinic polymorph [Booysen *et al.* (2011 ▶). *Acta Cryst.* E**67**, o1592]. An intra­molecular N—H⋯S hydrogen bond is observed. In the crystal, inter­molecular N—H⋯O hydrogen bonds link the mol­ecules into zigzag chains along the *b* axis. The closest distance between the centroids of symmetry-related heterocyclic rings is 3.5161 (6) Å.

## Related literature

For background to chelating ligands, see: Gade (1998 ▶). For the crystal structure of the title compound in the same space group but different cell parameters, see: Booysen *et al.* (2011 ▶). For the crystal structures of other Schiff-bases derived from *ortho*-(thio­meth­yl)-benzaldehyde, see: Yan *et al.* (2007 ▶); Baidina *et al.* (1987 ▶). For graph-set analysis of hydrogen bonds, see: Etter *et al.* (1990 ▶); Bernstein *et al.* (1995 ▶). For puckering analysis, see: Cremer & Pople (1975 ▶).
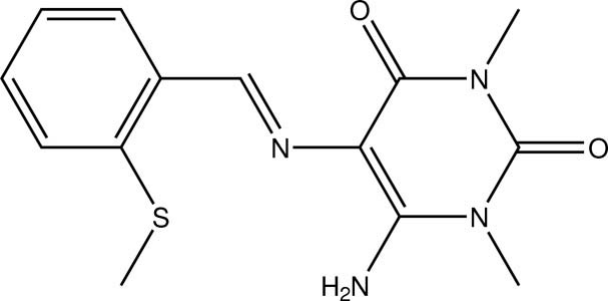


## Experimental

### 

#### Crystal data

C_14_H_16_N_4_O_2_S
*M* *_r_* = 304.37
Monoclinic, 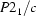

*a* = 13.5230 (2) Å
*b* = 13.8520 (3) Å
*c* = 7.5180 (1) Å
β = 101.068 (1)°
*V* = 1382.08 (4) Å^3^
*Z* = 4
Mo *K*α radiation
μ = 0.25 mm^−1^
*T* = 100 K
0.46 × 0.29 × 0.14 mm

#### Data collection

Bruker APEXII CCD diffractometer
13030 measured reflections
3420 independent reflections
3112 reflections with *I* > 2σ(*I*)
*R*_int_ = 0.029

#### Refinement

*R*[*F* ^2^ > 2σ(*F* ^2^)] = 0.029
*wR*(*F* ^2^) = 0.081
*S* = 1.02
3420 reflections
201 parameters
H atoms treated by a mixture of independent and constrained refinement
Δρ_max_ = 0.33 e Å^−3^
Δρ_min_ = −0.24 e Å^−3^

### 

Data collection: *APEX2* (Bruker, 2010 ▶); cell refinement: *SAINT* (Bruker, 2010 ▶); data reduction: *SAINT*; program(s) used to solve structure: *SIR97* (Altomare *et al.*, 1999 ▶); program(s) used to refine structure: *SHELXL97* (Sheldrick, 2008 ▶); molecular graphics: *ORTEP-3* (Farrugia, 1997 ▶) and *Mercury* (Macrae *et al.*, 2008 ▶); software used to prepare material for publication: *SHELXL97* and *PLATON* (Spek, 2009 ▶).

## Supplementary Material

Crystal structure: contains datablock(s) I, global. DOI: 10.1107/S1600536811027322/ci5192sup1.cif

Supplementary material file. DOI: 10.1107/S1600536811027322/ci5192Isup2.cdx

Structure factors: contains datablock(s) I. DOI: 10.1107/S1600536811027322/ci5192Isup3.hkl

Supplementary material file. DOI: 10.1107/S1600536811027322/ci5192Isup4.cml

Additional supplementary materials:  crystallographic information; 3D view; checkCIF report

## Figures and Tables

**Table 1:** Hydrogen-bond geometry (Å, °)

*D*—H⋯*A*	*D*—H	H⋯*A*	*D*⋯*A*	*D*—H⋯*A*
N4—H741⋯S1	0.83 (2)	2.675 (17)	3.5060 (10)	176 (1)
N4—H742⋯O1^i^	0.86 (2)	2.058 (16)	2.8797 (12)	160 (1)

Symmetry code: (i) 
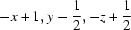
.

**Fig. 1.**
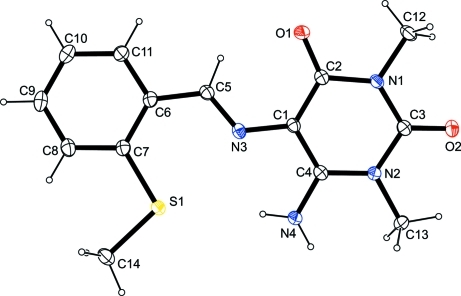
The molecular structure of the title compound, with atom labels and anisotropic displacement ellipsoids (drawn at the 50% probability level).

**Fig. 2.**
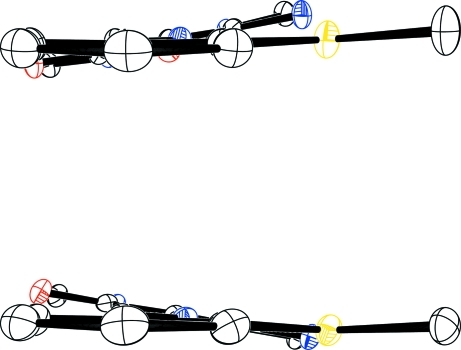
Comparison between the relative orientation of the planes of the non-aromatic six-membered ring with respect to the plane of the aromatic system in the molecular structure of the title compound determined earlier by us (Booysen et al., 2011, top) and in the present study (bottom). Hydrogen atoms were omitted for reasons of clarity.

**Fig. 3.**
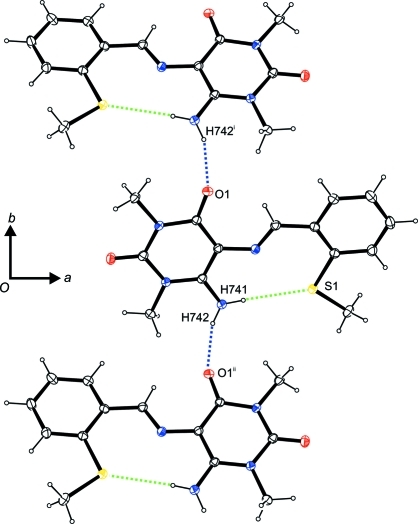
Intermolecular contacts, viewed along [001]. Green dashed lines indicate intramolecular hydrogen bonds and blue dashed lines intermolecular hydrogen bonds. Symmetry operators: (i) 1-x, 1/2+y, 1/2-z; (ii) 1-x, y-1/2, 1/2-z.

**Fig. 4.**
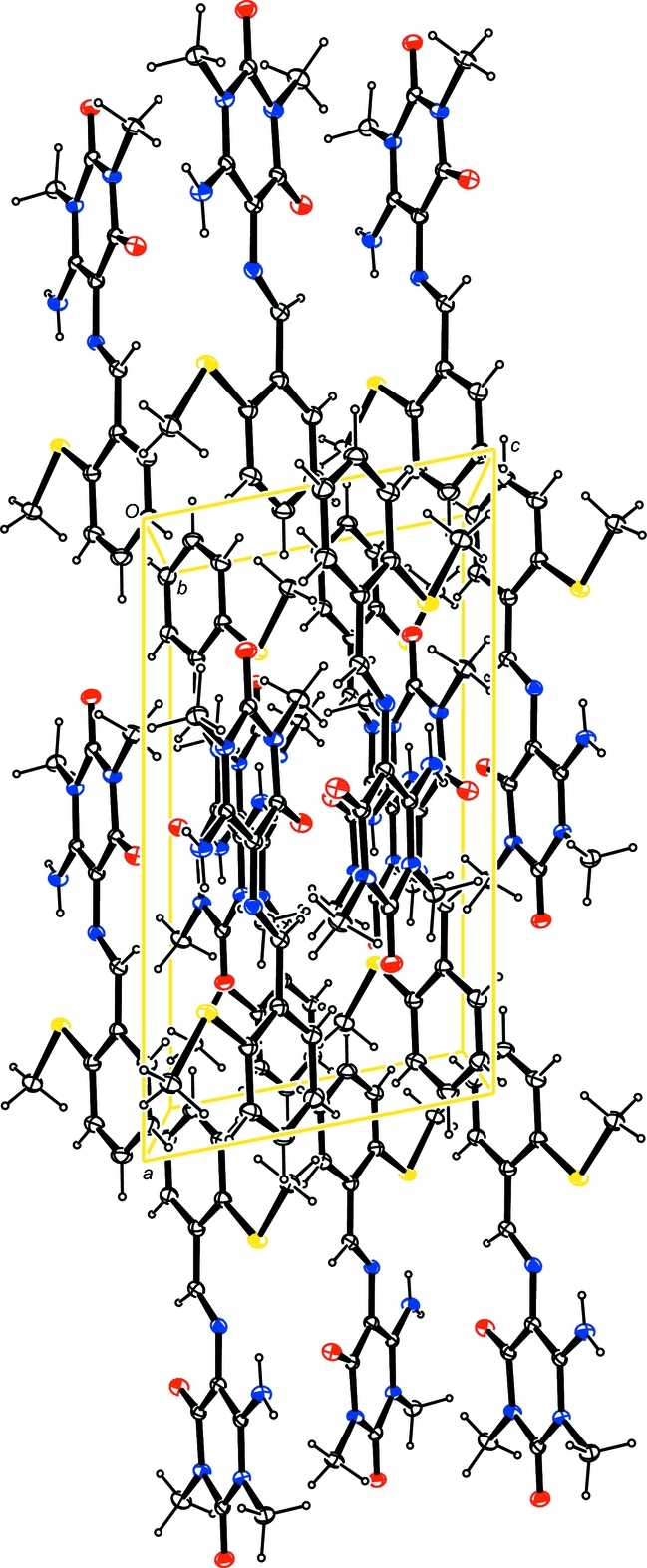
Molecular packing of the title compound, viewed along [010] (anisotropic displacement ellipsoids drawn at the 50% probability level).

## supplementary crystallographic information

### Comment

Chelate ligands have found widespread use in coordination chemistry due to the enhanced thermodynamic stability of resultant coordination compounds in relation to coordination compounds exclusively applying comparable monodentate ligands (Gade, 1998). Combining different sets of donor atoms in one chelate ligand molecule, a probe for testing and accomodating metal centers of different Lewis acidities is at hand. Recently, we performed a coordination reaction applying the title compound as the ligand. A single-crystal X-ray analysis of the isolated reaction product did not show the expected compound but the organic starting material whose crystal structure had been determined earlier by us in the same space-group, however, with different cell constants (Booysen *et al.*, 2011). Other crystal structures of Schiff-bases derived from *ortho*-(thiomethyl)-benzaldehyde are reported in the literature (Yan *et al.*, 2007; Baidina *et al.* 1987).

The molecule is a Schiff-base featuring an *ortho*-(thiomethyl)phenyl moiety and a 6-amino-1,3-dimethylpyrimidine-2,4(1*H*,3*H*)-dione moiety. The double-bond is (*E*)-configured. A conformational analysis of the non-aromatic six-membered ring (Cremer & Pople, 1975) fails due to the low puckering amplitude. The molecule is almost planar, the least-squares planes defined by the respective atoms of both six-membered ring systems intersect at an angle of only 8.77 (5) °. It is pertinent to emphasize that the plane of the non-aromatic ring is tilted in a different direction with respect to the molecular structure of the title compound determined earlier by us (Fig. 1 and Fig 2).

In the crystal structure, intra- as well as intermolecular hydrogen bonds can be observed, both supported by the amino group. While the intramolecular hydrogen bond is formed to the sulfur atom of the thiomethyl group, the intermolecular hydrogen bond uses one of the ketonic oxygen atoms as acceptor. The latter ones connect the molecules to zigzag chains along the crystallographic *b* axis. Unlike our earlier structure determination of the title compound, no C—H···O contacts are obvious. The descriptor for the hydrogen bonding system in terms of graph-set analysis (Etter *et al.*, 1990; Bernstein *et al.*, 1995) is *DC*^1^_1_(6) on the unitary level (Fig. 3). The shortest distance between the centroids of symmetry related heterocyclic rings was found to be 3.5161 (6) Å. The packing of the title compound is shown in Fig. 4.

### Experimental

The title compound was prepared as described in the literature (Booysen *et al.*, 2011). Single crystals suitable for the X-ray analysis were obtained by reacting the title compound with ReOBr_3_(PPh_3_)_2_ in methanol and subsequent free evaporation of the solvent.

### Refinement

H atoms bonded to aromatic C atoms were placed in calculated positions (C–H = 0.95 Å) and were included in the refinement in the riding model approximation, with *U*(H) set to 1.2*U*_eq_(C). The H  atoms of the methyl groups were allowed to rotate with a fixed angle around the C—C bond to best fit the experimental electron density (HFIX 137 in the *SHELX* program suite (Sheldrick, 2008)), with *U*(H) set to 1.5*U*_eq_(C). Both nitrogen-bound H atoms were located in a difference Fourier map and refined freely.

### Crystal data

**Table:**

C_14_H_16_N_4_O_2_S	*F*(000) = 640
*M**_r_* = 304.37	*D*_x_ = 1.463 Mg m^−^^3^
Monoclinic, *P*2_1_/*c*	Mo *K*α radiation, λ = 0.71069 Å
Hall symbol: -P 2ybc	Cell parameters from 9061 reflections
*a* = 13.5230 (2) Å	θ = 3.1–28.3°
*b* = 13.8520 (3) Å	µ = 0.25 mm^−^^1^
*c* = 7.5180 (1) Å	*T* = 100 K
β = 101.068 (1)°	Rod, orange
*V* = 1382.08 (4) Å^3^	0.46 × 0.29 × 0.14 mm
*Z* = 4	

### Data collection

**Table:**

Bruker APEXII CCD diffractometer	3112 reflections with *I* > 2σ(*I*)
Radiation source: fine-focus sealed tube	*R*_int_ = 0.029
graphite	θ_max_ = 28.4°, θ_min_ = 2.1°
φ and ω scans	*h* = −17→18
13030 measured reflections	*k* = −18→18
3420 independent reflections	*l* = −10→10

### Refinement

**Table:**

Refinement on *F*^2^	Primary atom site location: structure-invariant direct methods
Least-squares matrix: full	Secondary atom site location: difference Fourier map
*R*[*F*^2^ > 2σ(*F*^2^)] = 0.029	Hydrogen site location: inferred from neighbouring sites
*wR*(*F*^2^) = 0.081	H atoms treated by a mixture of independent and constrained refinement
*S* = 1.02	*w* = 1/[σ^2^(*F*_o_^2^) + (0.0421*P*)^2^ + 0.5901*P*] where *P* = (*F*_o_^2^ + 2*F*_c_^2^)/3
3420 reflections	(Δ/σ)_max_ = 0.001
201 parameters	Δρ_max_ = 0.33 e Å^−^^3^
0 restraints	Δρ_min_ = −0.24 e Å^−^^3^

### Fractional atomic coordinates and isotropic or equivalent isotropic displacement parameters (Å^2^)

**Table:**

	*x*	*y*	*z*	*U*_iso_*/*U*_eq_	
S1	0.78695 (2)	−0.045540 (19)	0.18639 (4)	0.01558 (9)	
O1	0.51894 (6)	0.21801 (5)	0.44986 (11)	0.01675 (17)	
O2	0.23379 (6)	0.03778 (6)	0.29316 (11)	0.01758 (17)	
N1	0.37683 (7)	0.12677 (6)	0.36962 (12)	0.01399 (18)	
N2	0.38044 (7)	−0.02607 (7)	0.23551 (12)	0.01352 (18)	
N3	0.63701 (7)	0.06360 (7)	0.31269 (12)	0.01372 (18)	
N4	0.52995 (7)	−0.08772 (7)	0.17260 (13)	0.01549 (19)	
H741	0.5910 (12)	−0.0762 (11)	0.181 (2)	0.023 (4)*	
H742	0.5004 (12)	−0.1407 (12)	0.134 (2)	0.026 (4)*	
C1	0.53409 (8)	0.06569 (7)	0.31391 (14)	0.0131 (2)	
C2	0.48115 (8)	0.14156 (8)	0.38191 (14)	0.0131 (2)	
C3	0.32410 (8)	0.04571 (8)	0.29933 (14)	0.0140 (2)	
C4	0.48168 (8)	−0.01659 (8)	0.23983 (13)	0.0128 (2)	
C5	0.69984 (8)	0.12764 (8)	0.38818 (14)	0.0144 (2)	
H5	0.6751	0.1802	0.4481	0.017*	
C6	0.80731 (8)	0.12361 (8)	0.38633 (14)	0.0145 (2)	
C7	0.85605 (8)	0.05250 (8)	0.29813 (14)	0.0147 (2)	
C8	0.95997 (9)	0.06045 (8)	0.30539 (16)	0.0182 (2)	
H8	0.9931	0.0137	0.2453	0.022*	
C9	1.01545 (9)	0.13518 (9)	0.39841 (16)	0.0203 (2)	
H9	1.0858	0.1393	0.4006	0.024*	
C10	0.96888 (9)	0.20383 (9)	0.48817 (16)	0.0195 (2)	
H10	1.0070	0.2545	0.5535	0.023*	
C11	0.86603 (8)	0.19752 (8)	0.48140 (15)	0.0170 (2)	
H11	0.8342	0.2447	0.5430	0.020*	
C12	0.31814 (9)	0.20105 (8)	0.44153 (16)	0.0183 (2)	
H121	0.2763	0.2358	0.3408	0.027*	
H122	0.3640	0.2465	0.5159	0.027*	
H123	0.2748	0.1705	0.5160	0.027*	
C13	0.32800 (8)	−0.11442 (8)	0.16217 (15)	0.0166 (2)	
H131	0.3329	−0.1216	0.0345	0.025*	
H132	0.2569	−0.1104	0.1722	0.025*	
H133	0.3591	−0.1703	0.2309	0.025*	
C14	0.87950 (9)	−0.10803 (9)	0.08635 (16)	0.0199 (2)	
H141	0.9071	−0.0639	0.0063	0.030*	
H142	0.8478	−0.1632	0.0160	0.030*	
H143	0.9340	−0.1310	0.1823	0.030*	

### Atomic displacement parameters (Å^2^)

**Table:**

	*U*^11^	*U*^22^	*U*^33^	*U*^12^	*U*^13^	*U*^23^
S1	0.01406 (14)	0.01421 (14)	0.01947 (14)	0.00057 (9)	0.00576 (10)	−0.00138 (9)
O1	0.0172 (4)	0.0118 (4)	0.0220 (4)	−0.0002 (3)	0.0056 (3)	−0.0008 (3)
O2	0.0121 (4)	0.0204 (4)	0.0200 (4)	0.0009 (3)	0.0026 (3)	−0.0007 (3)
N1	0.0130 (4)	0.0124 (4)	0.0173 (4)	0.0021 (3)	0.0048 (3)	−0.0002 (3)
N2	0.0126 (4)	0.0124 (4)	0.0154 (4)	0.0001 (3)	0.0024 (3)	−0.0008 (3)
N3	0.0132 (4)	0.0141 (4)	0.0145 (4)	0.0015 (3)	0.0042 (3)	0.0026 (3)
N4	0.0138 (4)	0.0127 (4)	0.0203 (4)	0.0006 (4)	0.0041 (4)	−0.0022 (3)
C1	0.0129 (5)	0.0127 (5)	0.0140 (4)	0.0013 (4)	0.0035 (4)	0.0017 (4)
C2	0.0136 (5)	0.0130 (5)	0.0131 (4)	0.0017 (4)	0.0036 (4)	0.0030 (4)
C3	0.0144 (5)	0.0147 (5)	0.0127 (5)	0.0025 (4)	0.0018 (4)	0.0024 (4)
C4	0.0138 (5)	0.0132 (5)	0.0114 (4)	0.0026 (4)	0.0026 (4)	0.0026 (4)
C5	0.0151 (5)	0.0133 (5)	0.0156 (5)	0.0013 (4)	0.0049 (4)	0.0016 (4)
C6	0.0148 (5)	0.0137 (5)	0.0152 (5)	−0.0004 (4)	0.0041 (4)	0.0036 (4)
C7	0.0149 (5)	0.0139 (5)	0.0157 (5)	−0.0001 (4)	0.0038 (4)	0.0032 (4)
C8	0.0156 (5)	0.0189 (5)	0.0214 (5)	0.0011 (4)	0.0066 (4)	0.0034 (4)
C9	0.0135 (5)	0.0243 (6)	0.0235 (5)	−0.0030 (4)	0.0044 (4)	0.0054 (5)
C10	0.0181 (5)	0.0198 (5)	0.0200 (5)	−0.0058 (4)	0.0019 (4)	0.0023 (4)
C11	0.0171 (5)	0.0152 (5)	0.0192 (5)	−0.0011 (4)	0.0045 (4)	0.0005 (4)
C12	0.0178 (5)	0.0158 (5)	0.0233 (5)	0.0044 (4)	0.0088 (4)	−0.0008 (4)
C13	0.0147 (5)	0.0141 (5)	0.0205 (5)	−0.0019 (4)	0.0021 (4)	−0.0022 (4)
C14	0.0185 (5)	0.0208 (5)	0.0216 (5)	0.0046 (4)	0.0065 (4)	−0.0021 (4)

### Geometric parameters (Å, °)

**Table:**

S1—C7	1.7669 (11)	C6—C11	1.4041 (15)
S1—C14	1.8008 (11)	C6—C7	1.4184 (15)
O1—C2	1.2423 (13)	C7—C8	1.4003 (15)
O2—C3	1.2182 (13)	C8—C9	1.3863 (17)
N1—C3	1.3799 (14)	C8—H8	0.95
N1—C2	1.4108 (13)	C9—C10	1.3856 (17)
N1—C12	1.4648 (13)	C9—H9	0.95
N2—C4	1.3694 (13)	C10—C11	1.3847 (15)
N2—C3	1.3927 (13)	C10—H10	0.95
N2—C13	1.4679 (13)	C11—H11	0.95
N3—C5	1.2829 (14)	C12—H121	0.98
N3—C1	1.3940 (13)	C12—H122	0.98
N4—C4	1.3339 (14)	C12—H123	0.98
N4—H741	0.832 (16)	C13—H131	0.98
N4—H742	0.859 (16)	C13—H132	0.98
C1—C4	1.3999 (14)	C13—H133	0.98
C1—C2	1.4209 (14)	C14—H141	0.98
C5—C6	1.4572 (15)	C14—H142	0.98
C5—H5	0.95	C14—H143	0.98
			
C7—S1—C14	102.80 (5)	C6—C7—S1	120.35 (8)
C3—N1—C2	125.45 (9)	C9—C8—C7	121.35 (10)
C3—N1—C12	115.86 (9)	C9—C8—H8	119.3
C2—N1—C12	118.66 (9)	C7—C8—H8	119.3
C4—N2—C3	122.27 (9)	C10—C9—C8	120.37 (10)
C4—N2—C13	119.84 (9)	C10—C9—H9	119.8
C3—N2—C13	117.89 (9)	C8—C9—H9	119.8
C5—N3—C1	124.05 (9)	C11—C10—C9	119.08 (11)
C4—N4—H741	112.5 (11)	C11—C10—H10	120.5
C4—N4—H742	122.0 (10)	C9—C10—H10	120.5
H741—N4—H742	125.4 (15)	C10—C11—C6	122.05 (10)
N3—C1—C4	114.23 (9)	C10—C11—H11	119.0
N3—C1—C2	126.19 (10)	C6—C11—H11	119.0
C4—C1—C2	119.58 (10)	N1—C12—H121	109.5
O1—C2—N1	118.55 (9)	N1—C12—H122	109.5
O1—C2—C1	125.63 (10)	H121—C12—H122	109.5
N1—C2—C1	115.82 (9)	N1—C12—H123	109.5
O2—C3—N1	121.91 (10)	H121—C12—H123	109.5
O2—C3—N2	122.23 (10)	H122—C12—H123	109.5
N1—C3—N2	115.86 (9)	N2—C13—H131	109.5
N4—C4—N2	118.68 (10)	N2—C13—H132	109.5
N4—C4—C1	120.32 (10)	H131—C13—H132	109.5
N2—C4—C1	121.00 (9)	N2—C13—H133	109.5
N3—C5—C6	123.11 (10)	H131—C13—H133	109.5
N3—C5—H5	118.4	H132—C13—H133	109.5
C6—C5—H5	118.4	S1—C14—H141	109.5
C11—C6—C7	118.44 (10)	S1—C14—H142	109.5
C11—C6—C5	115.77 (9)	H141—C14—H142	109.5
C7—C6—C5	125.79 (10)	S1—C14—H143	109.5
C8—C7—C6	118.69 (10)	H141—C14—H143	109.5
C8—C7—S1	120.94 (8)	H142—C14—H143	109.5
			
C5—N3—C1—C4	−173.63 (10)	C13—N2—C4—C1	−178.11 (9)
C5—N3—C1—C2	6.25 (17)	N3—C1—C4—N4	−1.25 (14)
C3—N1—C2—O1	−179.44 (9)	C2—C1—C4—N4	178.85 (9)
C12—N1—C2—O1	−1.49 (14)	N3—C1—C4—N2	178.43 (9)
C3—N1—C2—C1	0.49 (15)	C2—C1—C4—N2	−1.47 (15)
C12—N1—C2—C1	178.43 (9)	C1—N3—C5—C6	179.53 (9)
N3—C1—C2—O1	0.43 (17)	N3—C5—C6—C11	−177.63 (10)
C4—C1—C2—O1	−179.68 (10)	N3—C5—C6—C7	2.35 (17)
N3—C1—C2—N1	−179.49 (9)	C11—C6—C7—C8	−1.72 (15)
C4—C1—C2—N1	0.40 (14)	C5—C6—C7—C8	178.31 (10)
C2—N1—C3—O2	179.92 (9)	C11—C6—C7—S1	176.75 (8)
C12—N1—C3—O2	1.93 (15)	C5—C6—C7—S1	−3.23 (15)
C2—N1—C3—N2	−0.31 (15)	C14—S1—C7—C8	−5.40 (10)
C12—N1—C3—N2	−178.30 (9)	C14—S1—C7—C6	176.17 (9)
C4—N2—C3—O2	178.97 (10)	C6—C7—C8—C9	0.86 (16)
C13—N2—C3—O2	−1.22 (15)	S1—C7—C8—C9	−177.60 (9)
C4—N2—C3—N1	−0.80 (14)	C7—C8—C9—C10	0.49 (17)
C13—N2—C3—N1	179.01 (9)	C8—C9—C10—C11	−0.93 (17)
C3—N2—C4—N4	−178.61 (9)	C9—C10—C11—C6	0.01 (17)
C13—N2—C4—N4	1.58 (14)	C7—C6—C11—C10	1.31 (16)
C3—N2—C4—C1	1.70 (15)	C5—C6—C11—C10	−178.71 (10)

### Hydrogen-bond geometry (Å, °)

**Table:**

*D*—H···*A*	*D*—H	H···*A*	*D*···*A*	*D*—H···*A*
N4—H741···S1	0.83 (2)	2.675 (17)	3.5060 (10)	176 (1)
N4—H742···O1^i^	0.86 (2)	2.058 (16)	2.8797 (12)	160 (1)

Symmetry codes: (i) −*x*+1, *y*−1/2, −*z*+1/2.
